# Supplementary figures and images for: Endurance exercise ameliorates phenotypes in Drosophila models of spinocerebellar ataxias
Source: eLife. 2022 Feb 16;11:e75389. doi: 10.7554/eLife.75389 (PMC8871352; doi:10.7554/eLife.75389)

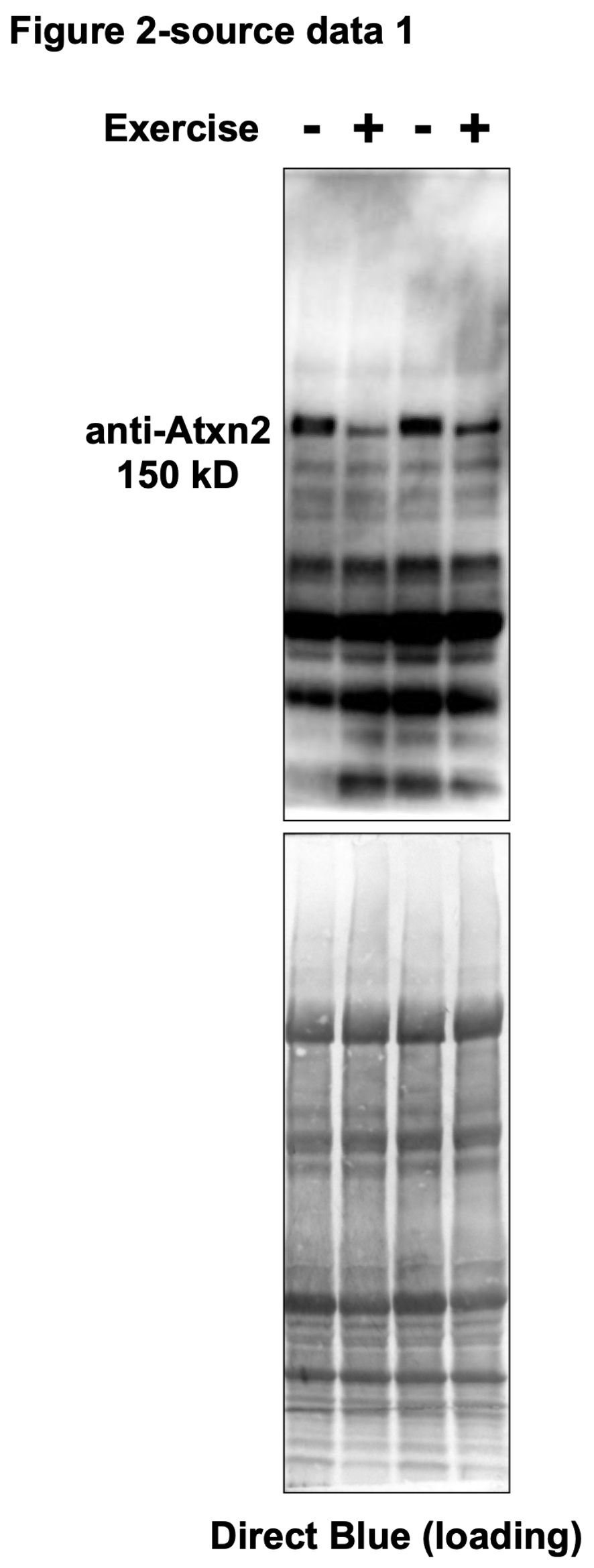

Supplement: Figure 2—source data 1. [file elife-75389-fig2-data1.zip › Figure 2-source data 1 figure.jpg]

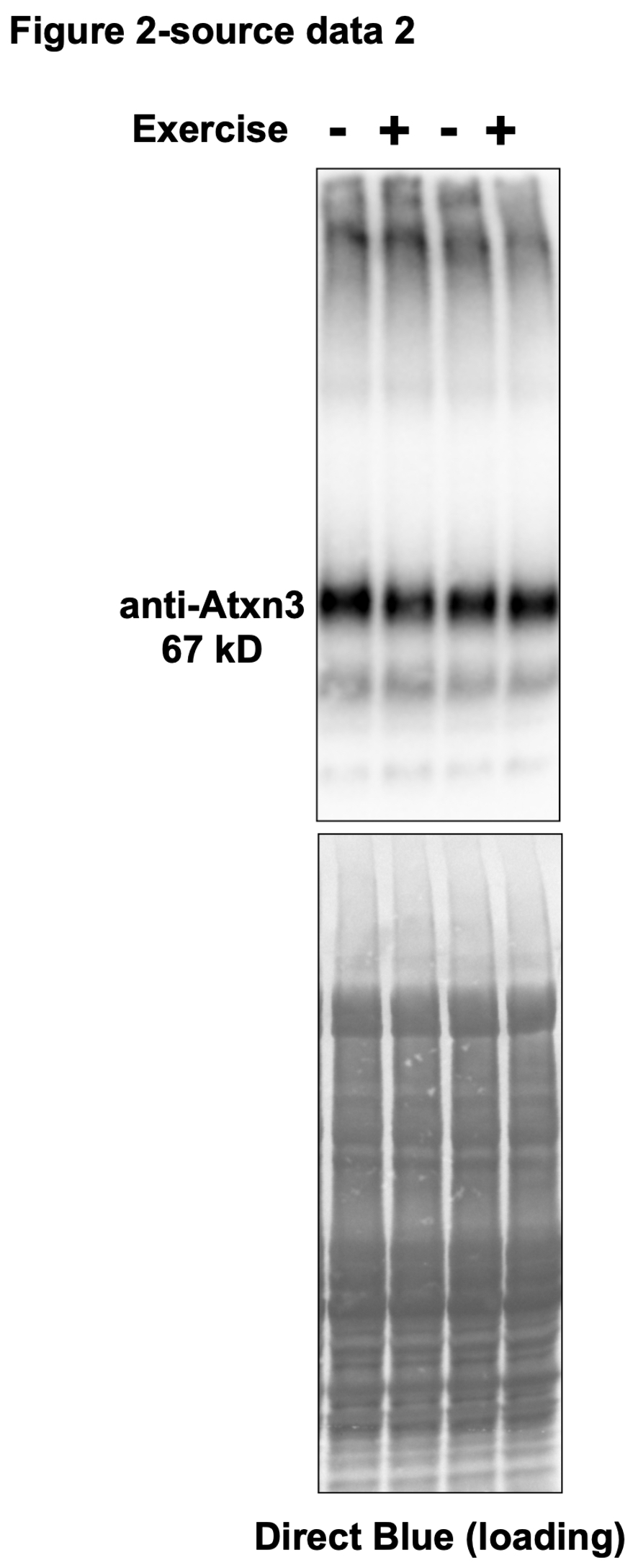

Supplement: Figure 2—source data 2. [file elife-75389-fig2-data2.zip › Figure 2-source data 2 figure.jpg]

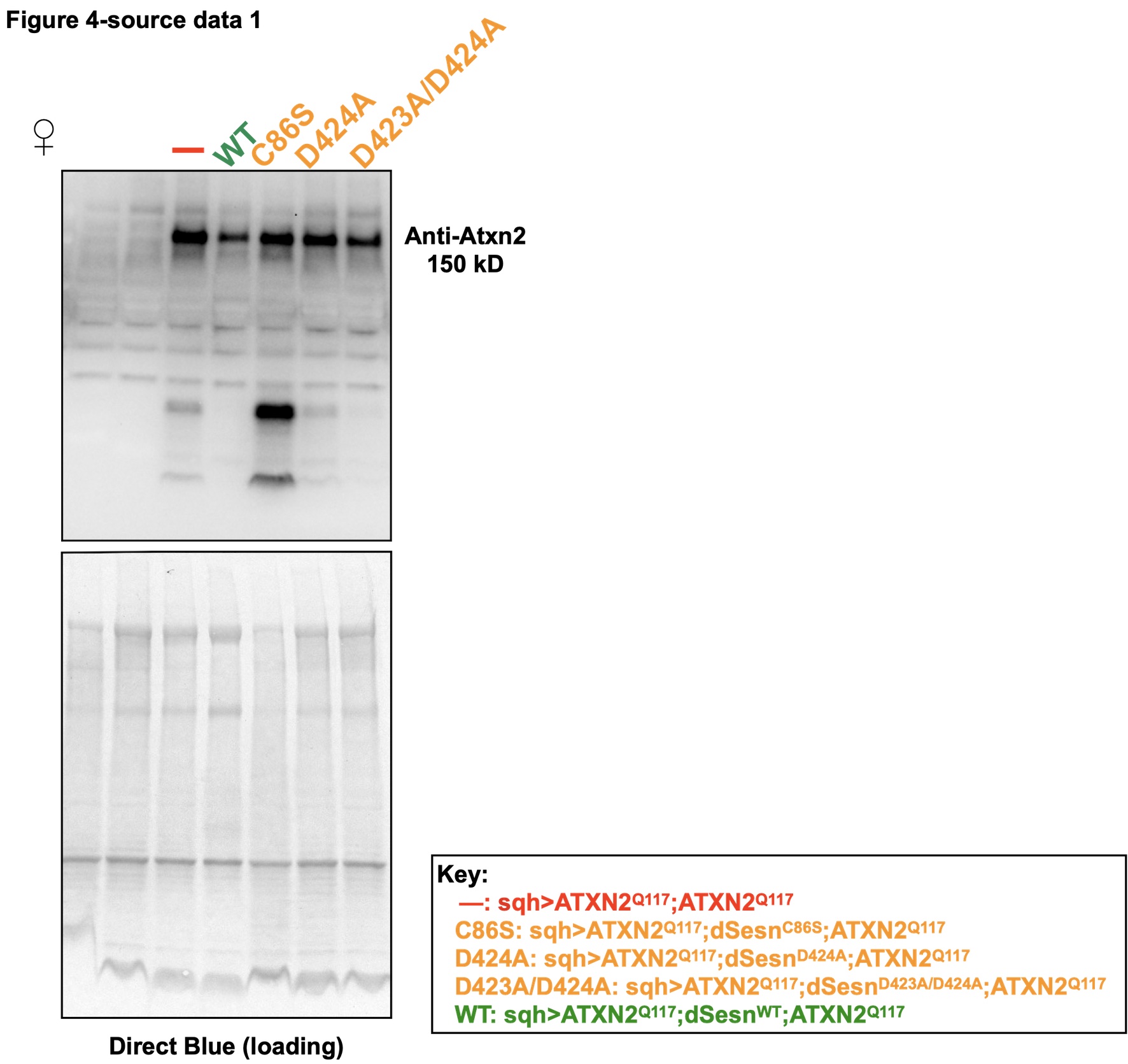

Supplement: Figure 4—source data 1. [file elife-75389-fig4-data1.zip › Figure 4-source data 1 figure.jpg]

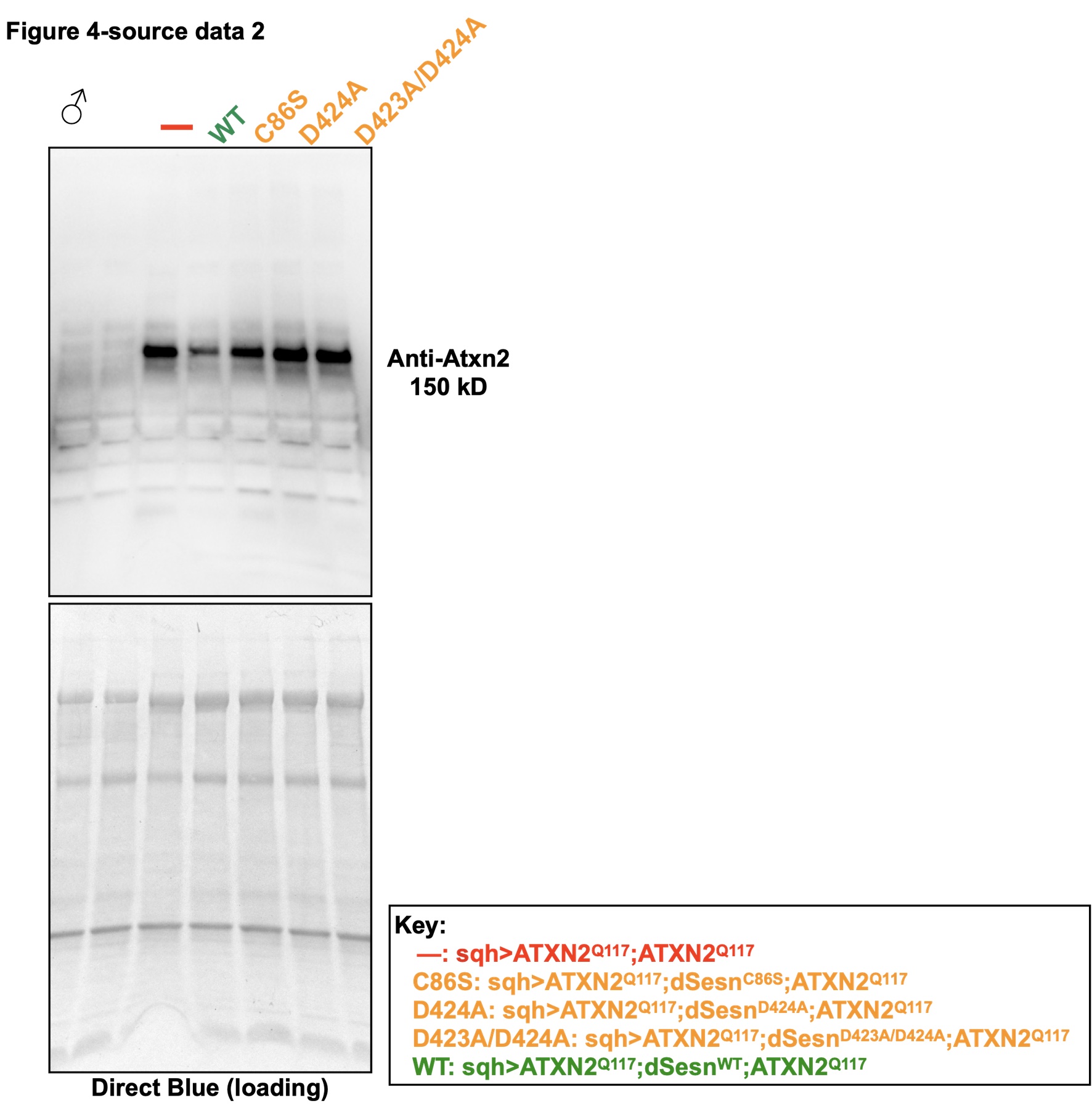

Supplement: Figure 4—source data 2. [file elife-75389-fig4-data2.zip › Figure 4-source data 2 figure.jpg]

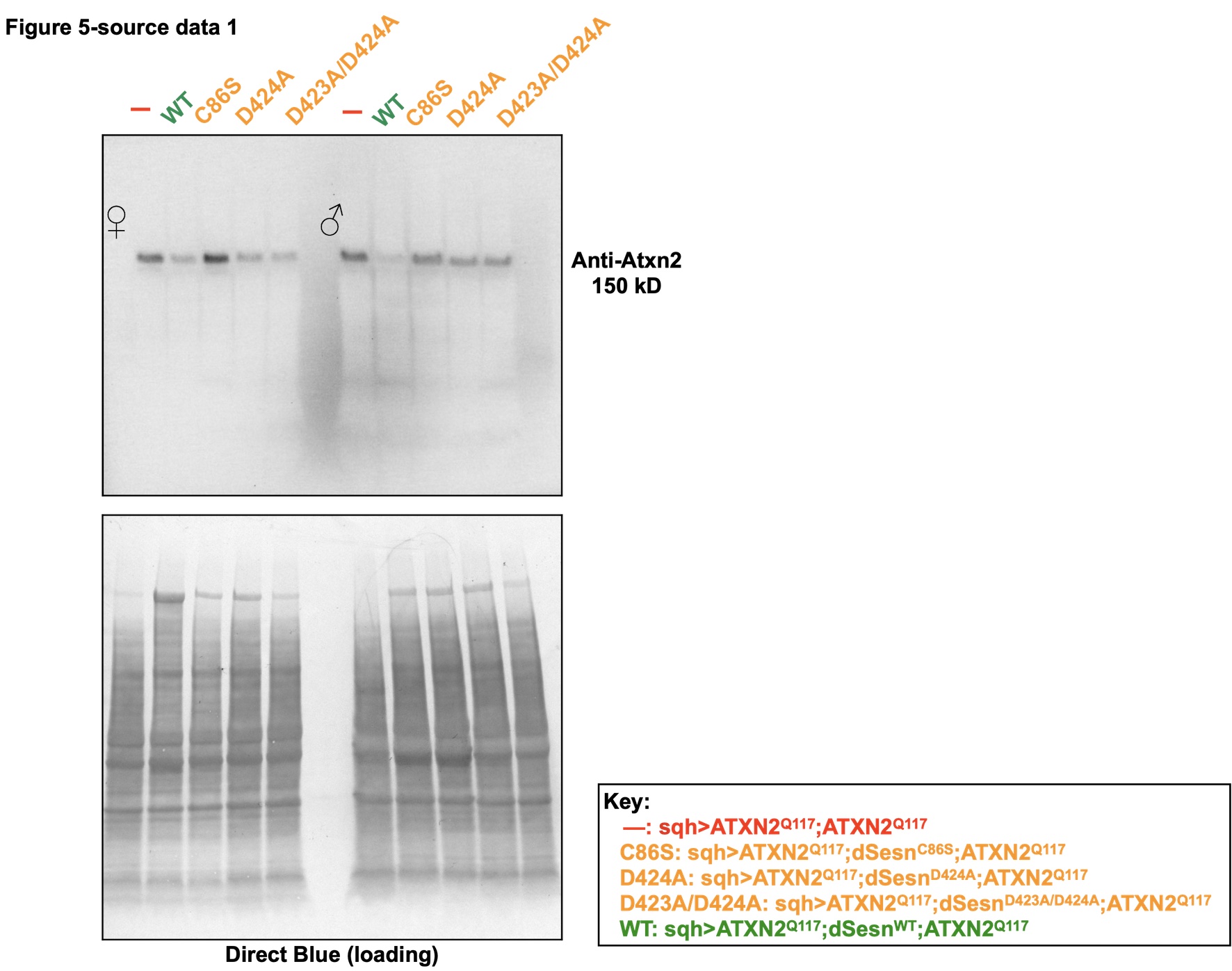

Supplement: Figure 5—source data 1. [file elife-75389-fig5-data1.zip › Figure 5-source data 1 figure.jpg]

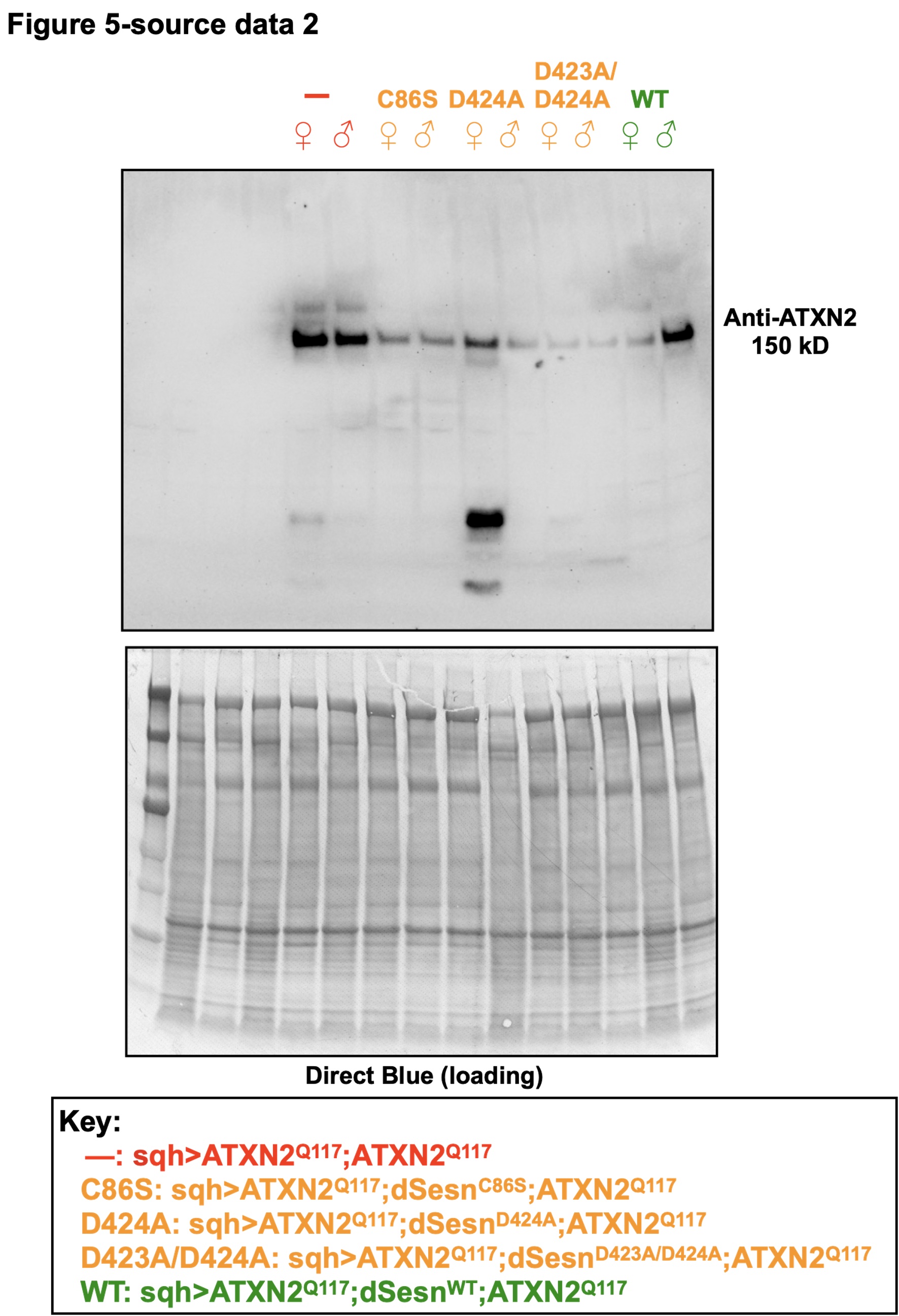

Supplement: Figure 5—source data 2. [file elife-75389-fig5-data2.zip › Figure 5-source data 2 figure.jpg]

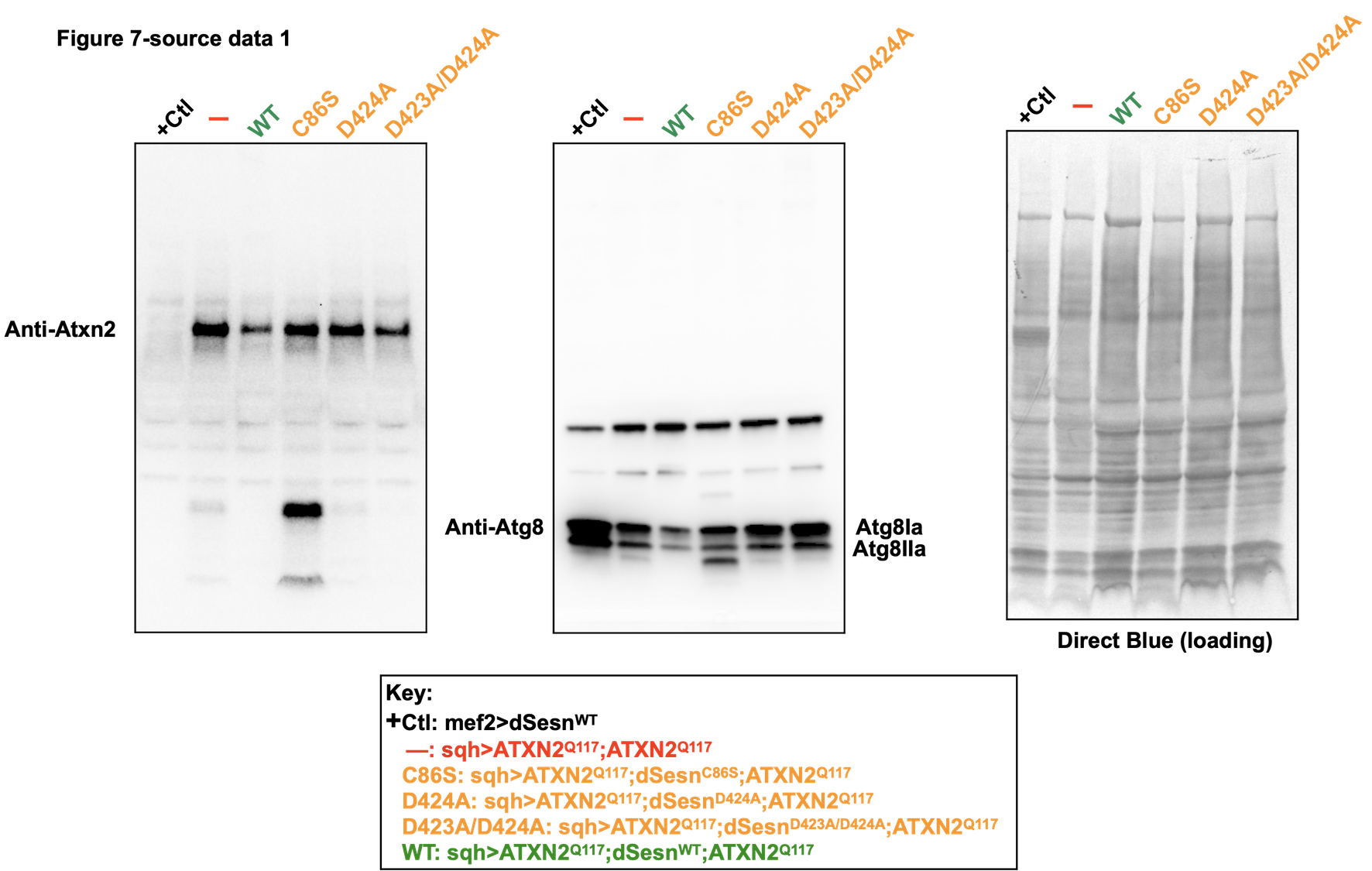

Supplement: Figure 7—source data 1. [file elife-75389-fig7-data1.zip › Figure 7-source data 1 figure.jpg]

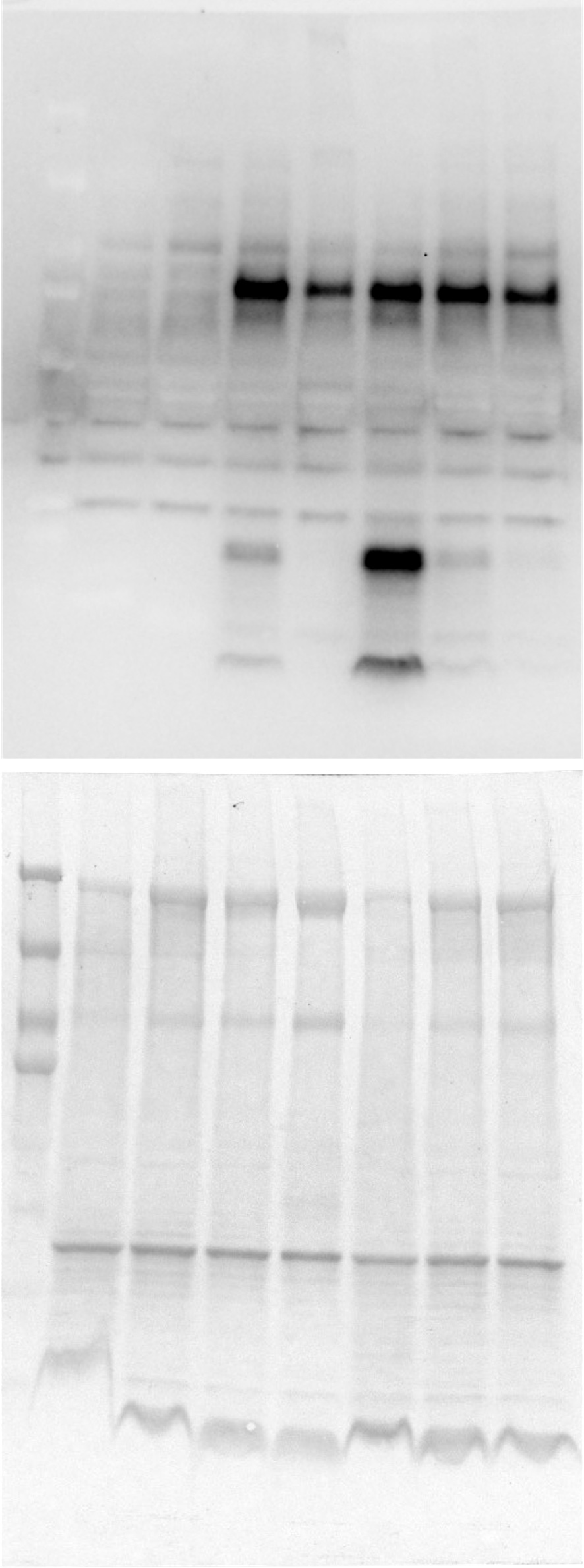

Supplement: Source data 1. [file elife-75389-data1.zip › 22_02_04_blot jpegs/Figure 4-source data 1.jpg]

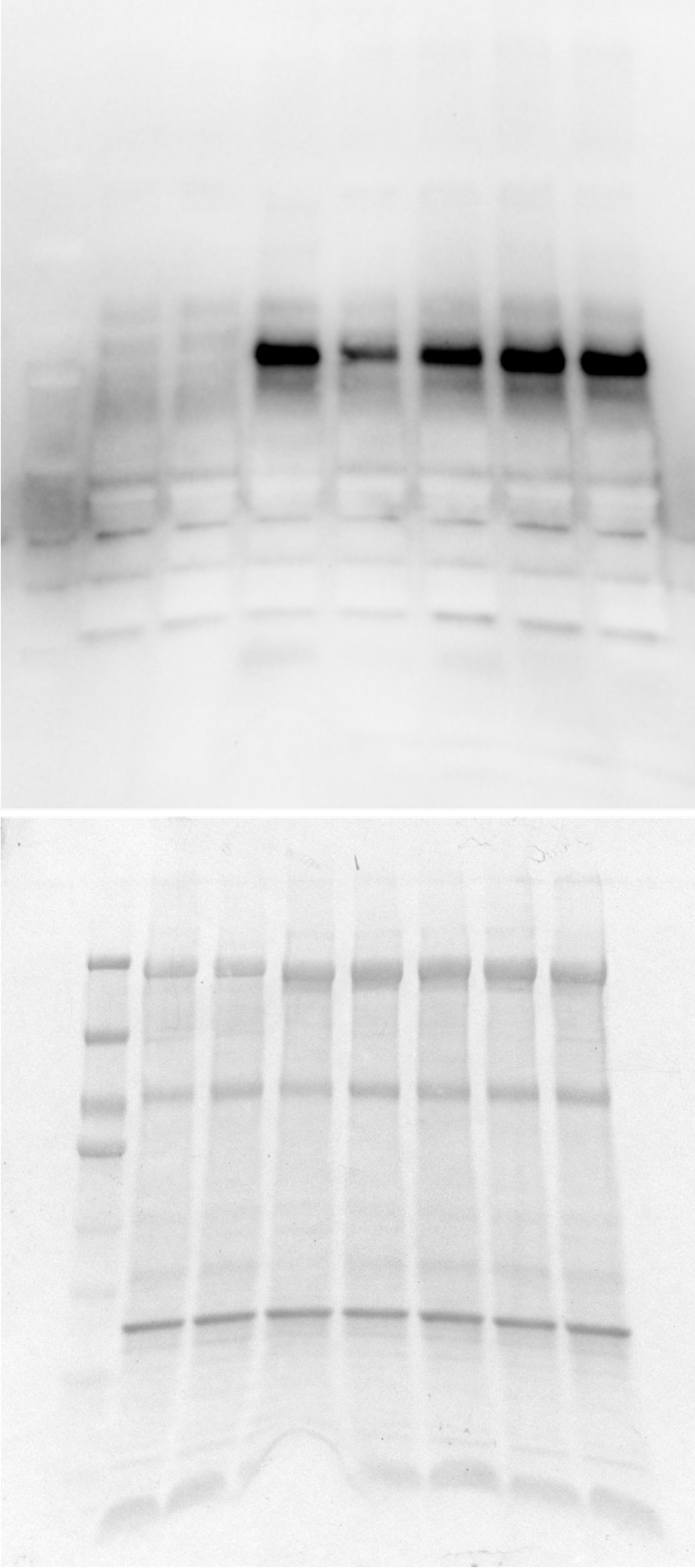

Supplement: Source data 1. [file elife-75389-data1.zip › 22_02_04_blot jpegs/Figure 4-source data 2.jpg]

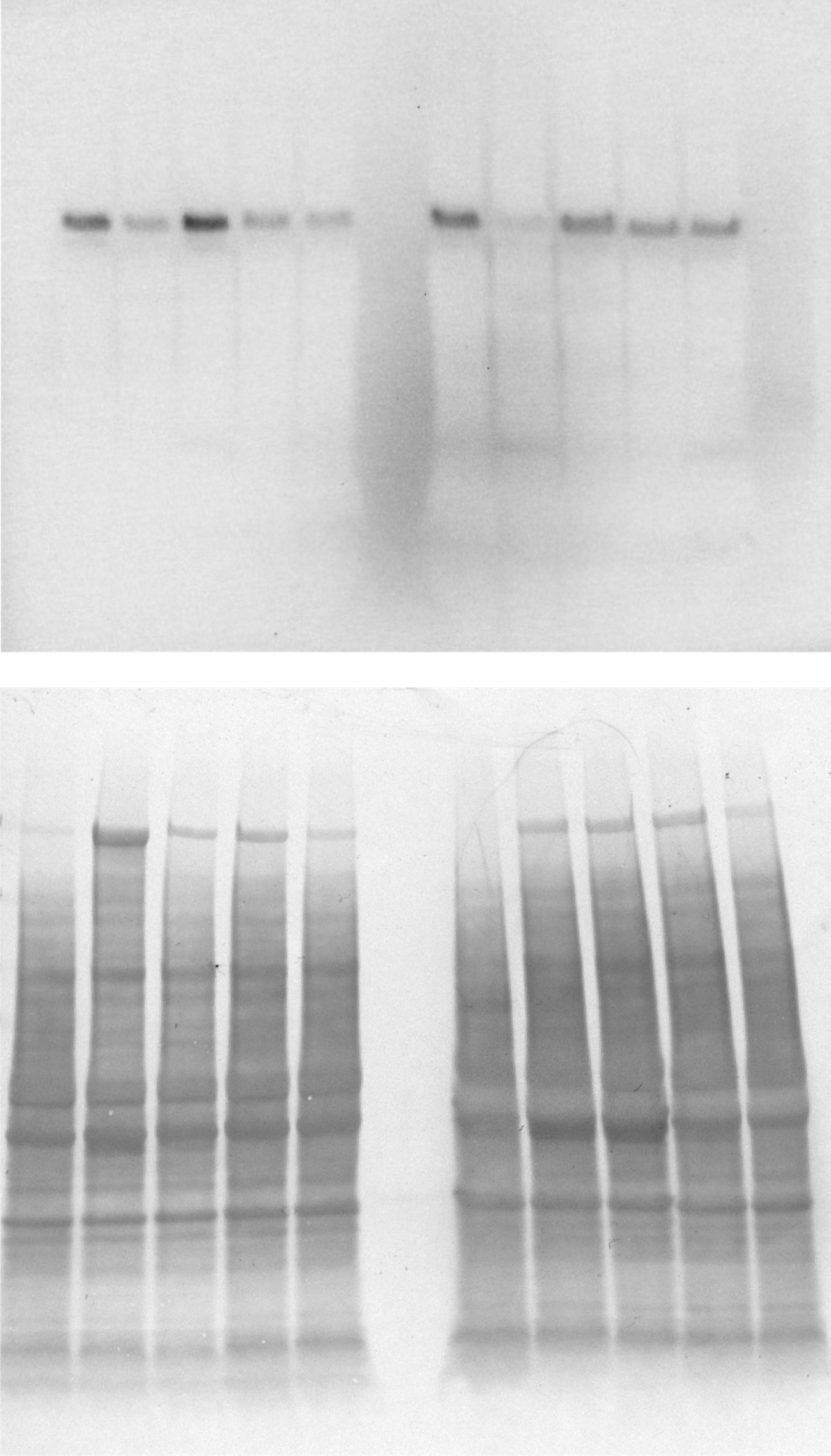

Supplement: Source data 1. [file elife-75389-data1.zip › 22_02_04_blot jpegs/Figure 5-source data 1.jpg]

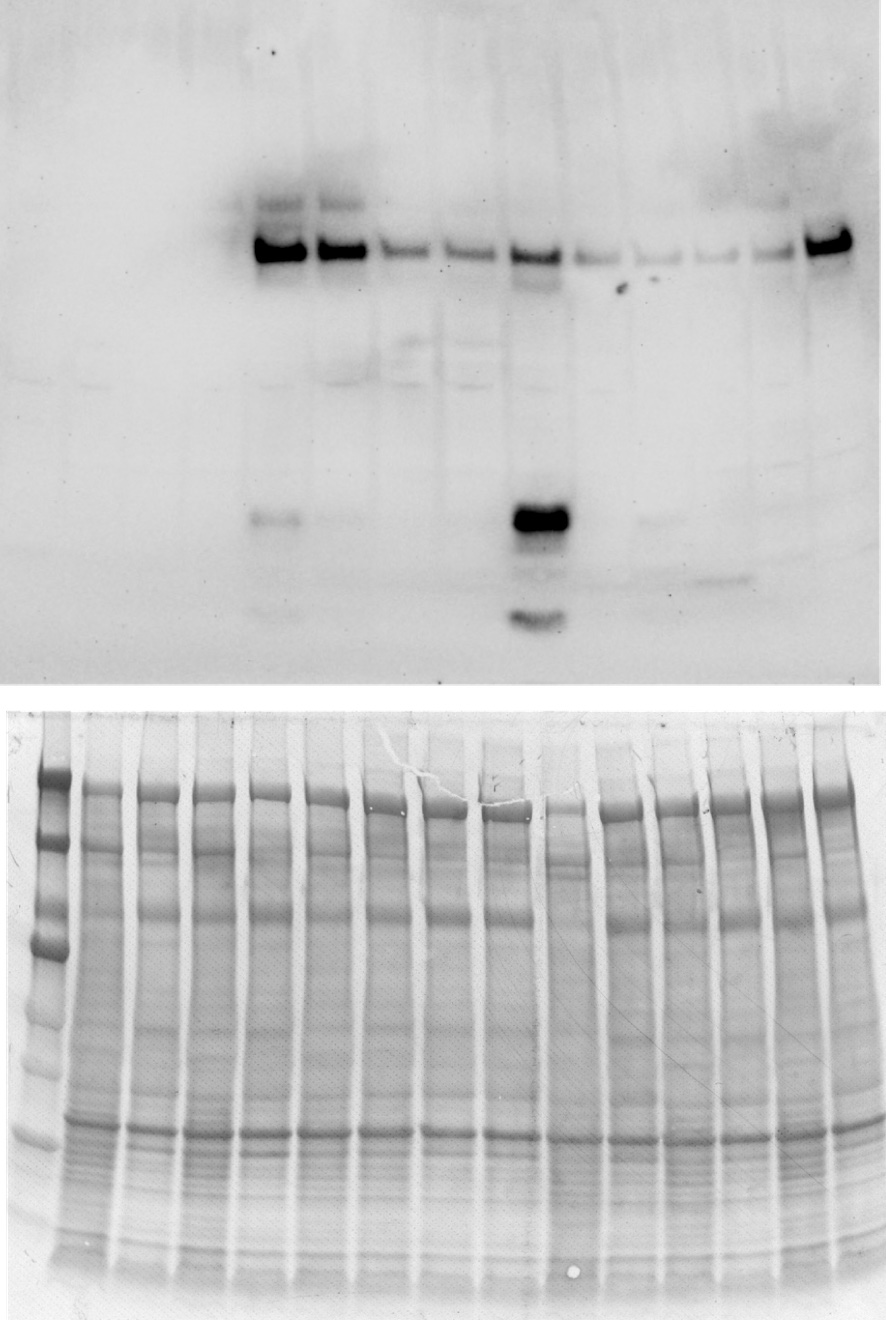

Supplement: Source data 1. [file elife-75389-data1.zip › 22_02_04_blot jpegs/Figure 5-source data 2.jpg]

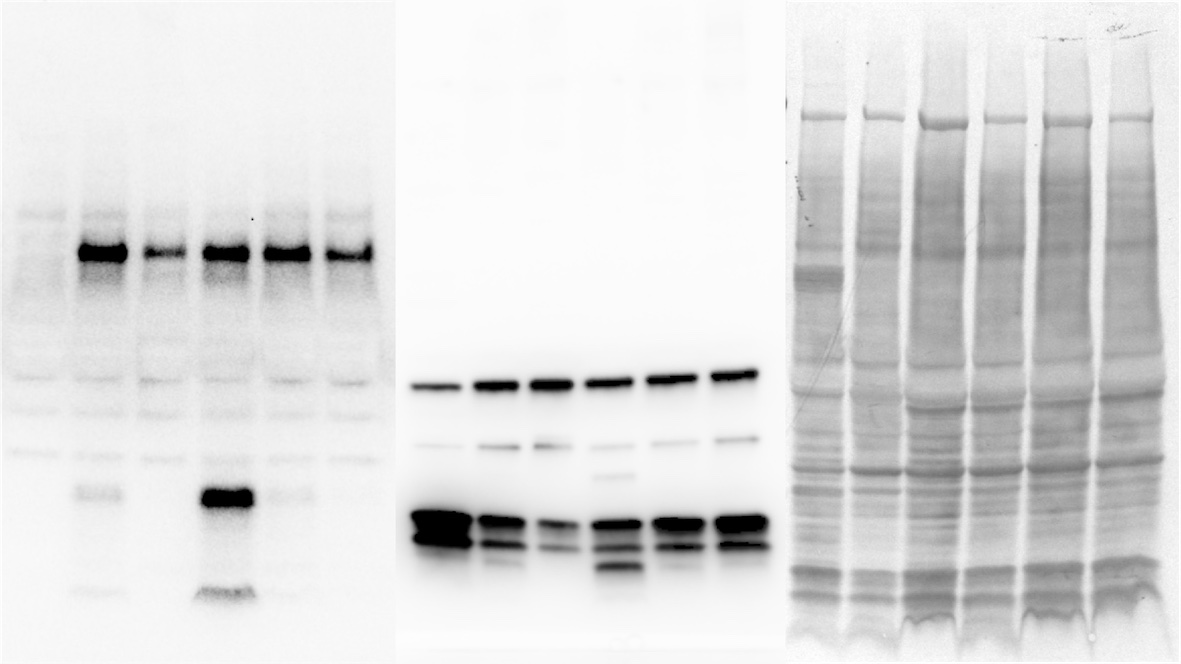

Supplement: Source data 1. [file elife-75389-data1.zip › 22_02_04_blot jpegs/Figure 7-source data 1.jpg]

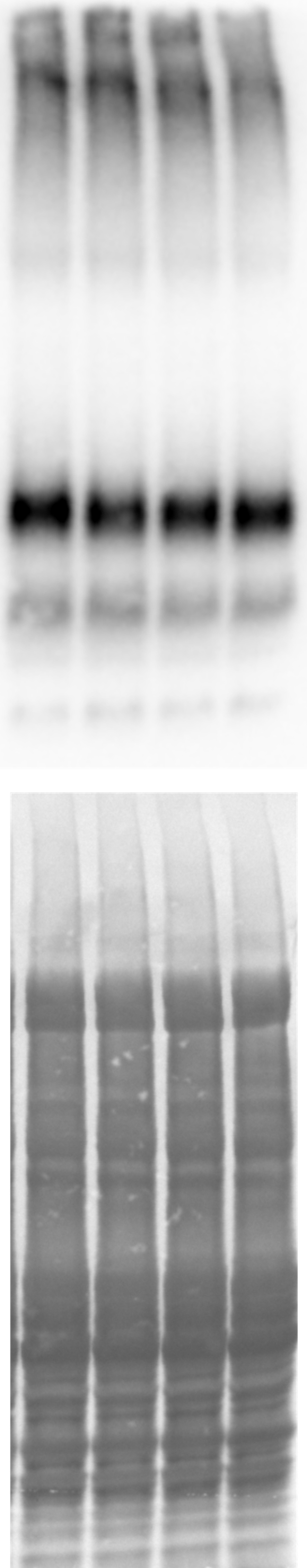

Supplement: Source data 1. [file elife-75389-data1.zip › 22_02_04_blot jpegs/Figure 2-source data 2.jpg]

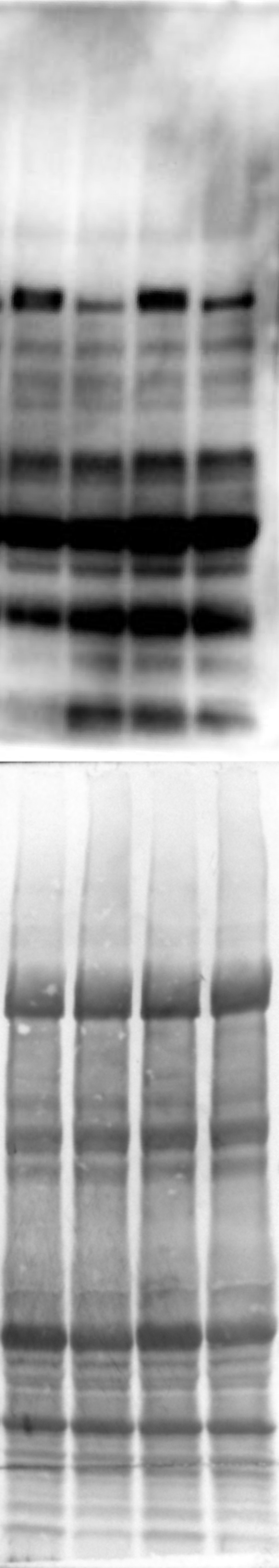

Supplement: Source data 1. [file elife-75389-data1.zip › 22_02_04_blot jpegs/Figure 2-source data 1.jpg]
