# Supplementary material for: Endurance exercise ameliorates phenotypes in Drosophila models of spinocerebellar ataxias
Source: eLife. 2022 Feb 16;11:e75389. doi: 10.7554/eLife.75389 (PMC8871352; doi:10.7554/eLife.75389)
Supplement: Transparent reporting form [file elife-75389-transrepform1.docx]

***eLife’s* transparent reporting form**

We encourage authors to provide detailed information *within their submission* to facilitate the interpretation and replication of experiments. Authors can upload supporting documentation to indicate the use of appropriate reporting guidelines for health-related research (see [EQUATOR Network](http://www.equator-network.org/%20)), life science research (see the [BioSharing Information Resource](https://biosharing.org/)), or the [ARRIVE guidelines](http://www.plosbiology.org/article/info:doi/10.1371/journal.pbio.1000412) for reporting work involving animal research. Where applicable, authors should refer to any relevant reporting standards documents in this form.

If you have any questions, please consult our Journal Policies and/or contact us: [editorial@elifesciences.org](mailto:editorial@elifesciences.org).

**Sample-size estimation**

- You should state whether an appropriate sample size was computed when the study was being designed
- You should state the statistical method of sample size computation and any required assumptions
- If no explicit power analysis was used, you should describe how you decided what sample (replicate) size (number) to use

Please outline where this information can be found within the submission (e.g., sections or figure legends), or explain why this information doesn’t apply to your submission:

Sample size for each experiment is listed in the legends, and exact sample sizes can be found in the source data file for each figure. Although no explicit power analyses were used to determine sample size, *Drosophila* lifespan and motility typically have large cohort sizes with impressive power. While an n of 50 is typically sufficient to observe differences between cohorts, no experiment reported an n of less than 100 individual flies, and all experiments were performed in both males and females. See Linford et al., *Measurement of lifespan in D. melanogaster*. [J Vis Exp.](https://www.ncbi.nlm.nih.gov/pmc/articles/PMC3582515/) 2013; (71): 50068. For additional description of appropriate experimental size.

**Replicates**

- You should report how often each experiment was performed
- You should include a definition of biological versus technical replication
- The data obtained should be provided and sufficient information should be provided to indicate the number of independent biological and/or technical replicates
- If you encountered any outliers, you should describe how these were handled
- Criteria for exclusion/inclusion of data should be clearly stated
- High-throughput sequence data should be uploaded before submission, with a private link for reviewers provided (these are available from both GEO and ArrayExpress)

Please outline where this information can be found within the submission (e.g., sections or figure legends), or explain why this information doesn’t apply to your submission:

Biological replicates of at least 2 cohorts of male and female flies were performed for all experiments in the main text, and repetitions are reported in the figure legends. Biological replicates consisted of separate fly cohorts for every experiment reported. Replicate blots are shown in the source data file in their uncropped and unedited form.

**Statistical reporting**

- Statistical analysis methods should be described and justified
- Raw data should be presented in figures whenever informative to do so (typically when N per group is less than 10)
- For each experiment, you should identify the statistical tests used, exact values of N, definitions of center, methods of multiple test correction, and dispersion and precision measures (e.g., mean, median, SD, SEM, confidence intervals; and, for the major substantive results, a measure of effect size (e.g., Pearson's r, Cohen's d)
- Report exact p-values wherever possible alongside the summary statistics and 95% confidence intervals. These should be reported for all key questions and not only when the p-value is less than 0.05.

Please outline where this information can be found within the submission (e.g., sections or figure legends), or explain why this information doesn’t apply to your submission:

Statistical analyses are defined in the methods, lines 423-428. Figures depict all individual p-values, and figure legends report statistical tests used and the minimum n value for each individual panel. Histograms include individual data points, and all raw data can be located in the source file.

(For large datasets, or papers with a very large number of statistical tests, you may upload a single table file with tests, Ns, etc., with reference to sections in the manuscript.)

**Group allocation**

- Indicate how samples were allocated into experimental groups (in the case of clinical studies, please specify allocation to treatment method); if randomization was used, please also state if restricted randomization was applied
- Indicate if masking was used during group allocation, data collection and/or data analysis

Please outline where this information can be found within the submission (e.g., sections or figure legends), or explain why this information doesn’t apply to your submission:

Group allocation is located in the methods section, lines 345-368.

**Additional data files (“source data”)**

- We encourage you to upload relevant additional data files, such as numerical data that are represented as a graph in a figure, or as a summary table
- Where provided, these should be in the most useful format, and they can be uploaded as “Source data” files linked to a main figure or table
- Include model definition files including the full list of parameters used
- Include code used for data analysis (e.g., R, MatLab)
- Avoid stating that data files are “available upon request”

Please indicate the figures or tables for which source data files have been provided:

Source data for all figures is included as a tab delimited excel file, which includes raw data and individual, uncropped blot repetitions.
